# Study of Platelet Parameters Among Dyslipidaemic Patients

**DOI:** 10.7759/cureus.98636

**Published:** 2025-12-07

**Authors:** Pavithraa Hariharan, Sri Gayathri Shanmugam, Nagarajan Priyathersini, Muthu Subramanian P S

**Affiliations:** 1 Department of Pathology, Sri Ramachandra Institute of Higher Education and Research, Chennai, IND

**Keywords:** dyslipidemia, lipid disoders, platelet activation, platelets function, thrombosis

## Abstract

Background

Platelets play a critical role in thrombotic events. Larger platelets are more enzymatically and metabolically active and therefore have a higher thrombotic potential than smaller platelets. Hyperlipidaemia can lead to various thromboembolic complications, and platelet parameters are useful for assessing this risk. Platelet volume indices are simple, inexpensive, and reliable tools that may be useful for predicting impending acute cardiovascular events. Hyperlipidemia is a major risk factor, and studies generally show increased platelet volume indices among dyslipidemic patients compared to those with a normal lipid profile. However, a significant gap remains, as most existing studies report only a general correlation between platelet volume indices and the presence of dyslipidemia. Few to no studies have specifically correlated individual platelet indices with individual biochemical lipid parameters. Furthermore, data establishing this association in the South indian cohort is limited.

Aim

The aim of this study is to investigate the association between platelet parameters, including platelet volume indices, and lipid profile.

Methods

This was a cross-sectional comparative study done over four months from July 2024 to October 2024 with a sample size of 120 participants. Samples collected for complete blood count were included in the study and analysed for platelet parameters. Patients below 20 years of age, samples with prolonged storage, known hereditary platelet disorders, thrombocytopenia, previous blood transfusion, history of diabetes, patients receiving hypolipidaemic or antiplatelet medication, and those presenting with any acute inflammatory condition were excluded. The blood samples were analysed for platelet parameters: mean platelet volume (MPV), plateletcrit (Pct), platelet distribution width (PDW), and platelet-large cell ratio (P-LCR). The results were correlated with biochemical lipid profile values. The individual correlation of platelet parameters with every lipid parameter was done with Pearson's correlation test.

Results

Platelet parameters, particularly P-LCR and MPV, were significantly higher among patients with abnormal lipid profiles. Furthermore, a significant positive correlation was observed between individual platelet indices and specific lipid parameters.

Conclusion

This study demonstrates that a simple first-line test, the complete blood count, may help identify individuals with abnormal lipid levels who are at risk of thrombotic events such as cardiovascular disease. The positive correlation observed between individual Platelet volume indices and specific lipid parameters suggests that these indices could serve as a cost-effective, easily accessible marker for tracking patients with abnormal lipid profiles, preventing any ischemic events, and thereby reducing mortality.

## Introduction

Dyslipidaemia, characterised by abnormal cholesterol and triglyceride levels, is a major risk factor for cardiovascular disease, which remains the most common cause of morbidity and mortality in India [1-6]. Early diagnosis and regular follow-up are essential.

Platelets are closely related to lipid metabolism in terms of their development, structure, metabolism, and signalling. The platelet lipidome is a complex collection of lipids derived from both exogenous (dietary) and endogenous (de novo) sources. Platelets and megakaryocytes can synthesise lipids de novo, and their lipid composition and function are regulated by the phosphatidylinositol 3-kinase (PI3K) pathway. Upon activation, platelet membrane lipids rearrange with phosphatidylserine signalling, creating a procoagulant surface. Lipids are converted into signalling molecules that influence platelet aggregation, shape change, secretion, and coagulation. Consequently, the lipid profile has a direct impact on platelet activation. Hyperlipidaemia primes platelets and enhances platelet activation, which is a major underlying mechanism in thrombosis [7,8].

Abnormal levels of blood lipids will cause fat deposits in the artery walls, which initiate complications inside the blood vessels. Thereby thrombus occludes a blood vessel and may lead to complications like myocardial infarction. Activated platelets are typically larger, a change that can be reflected in altered platelet volume indices [9-11]. The commonly used hypolipidemic drugs like statins are also found to have some synergistic effects on antiplatelet drugs [12]. The aim of this study was to investigate the association between platelet volume indices and lipid profile.

## Materials and methods

This was a cross-sectional comparative study conducted at Sri Ramachandra Institute of Higher Education and Research, Chennai, India, over a four-month period from July 2024 to October 2024. Ethical clearance was obtained from the Institutional Ethics Committee, Sri Ramachandra Institute of Higher Education and Research (CSP-MED/24/AUG/107/265). Informed consent was waived as the data were derived from a laboratory database.

Study population

The sample size of the study was 120. The participants were recruited using a consecutive sampling method. Biochemical data were collected from the laboratory information system database after obtaining

Among 120 patients, those with deranged lipid profiles based on National Cholesterol Education Program (NCEP) guidelines were included in the study group (n = 60), and age- and sex-matched participants with normal lipid profiles served as controls (n = 60).

Samples collected for complete blood count were included in the study and analysed for platelet parameters. Patients below 20 years of age, samples with prolonged storage, known hereditary platelet disorders, thrombocytopenia, previous blood transfusion, history of diabetes, patients receiving hypolipidaemic or antiplatelet medication, and those presenting with any acute inflammatory condition were excluded from the study.

The 120 participants were divided into two groups: (i) Study group comprising patients with abnormal lipid profiles (total cholesterol > 200 mg/dL, fasting triglycerides > 150 mg/dL, low-density lipoproteins > 100 mg/dL), and (ii) Control group comprising patients with normal lipid profiles

Study process

**Figure 1:**
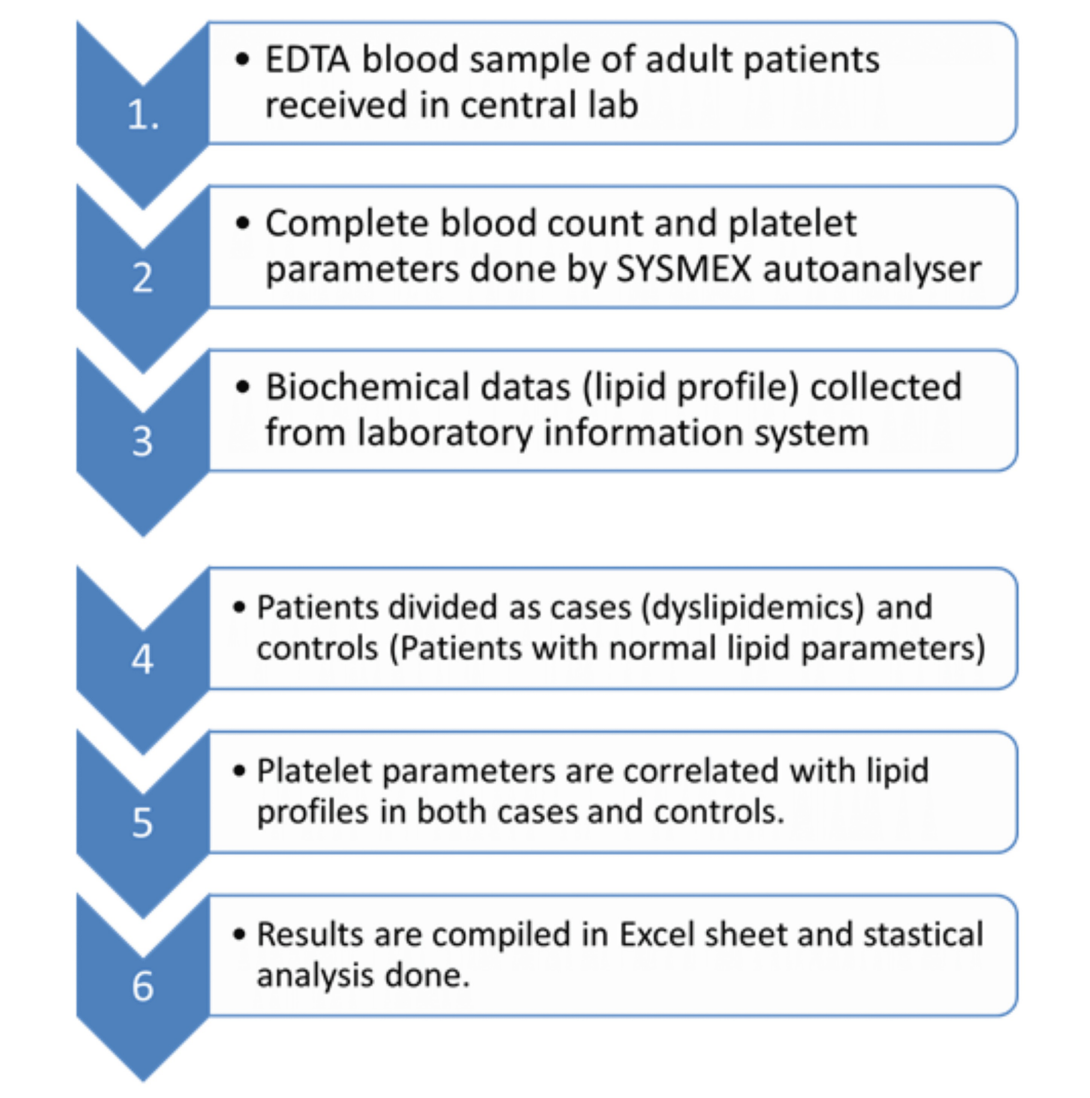
Flowchart representing the study process EDTA: ethylenediaminetetraacetic acid

Ethylenediaminetetraacetic acid (EDTA)-anticoagulated whole-blood samples were collected from all participants. Platelet parameters, including mean platelet volume (MPV), platelet large cell ratio (P-LCR), platelet distribution width (PDW), and plateletcrit (Pct), were analysed using the SYSMEX XN-3100 analyser (Sysmex Corporation, Kobe, Hyogo, Japan). Fasting blood samples were collected, and lipid profiling was done by the photoelectric method in cobas® e 602 (Roche Holding AG, Basel, Switzerland), and the data were obtained from the laboratory information system. The method of the study is explained as a flowchart (Figure 1).

Statistical analysis

The data were compiled in Microsoft Excel (Microsoft Corporation, Redmond, Washington, United States), and normality was analysed statistically using IBM SPSS Statistics for Windows, version 26 (IBM Corp., Armonk, New York, United States). The normality test was done by observing the QQ plot and the Kolmogorov-Smirnov test. A p-value of <0.05 in the Kolmogorov-Smirnov test was considered as non-normal data. As MPV was normally distributed, the mean values of the two groups were compared using the independent t-test. As the variable P-LCR was not normally distributed, the median was compared using the Mann-Whitney U test.

## Results

**Figure 2:**
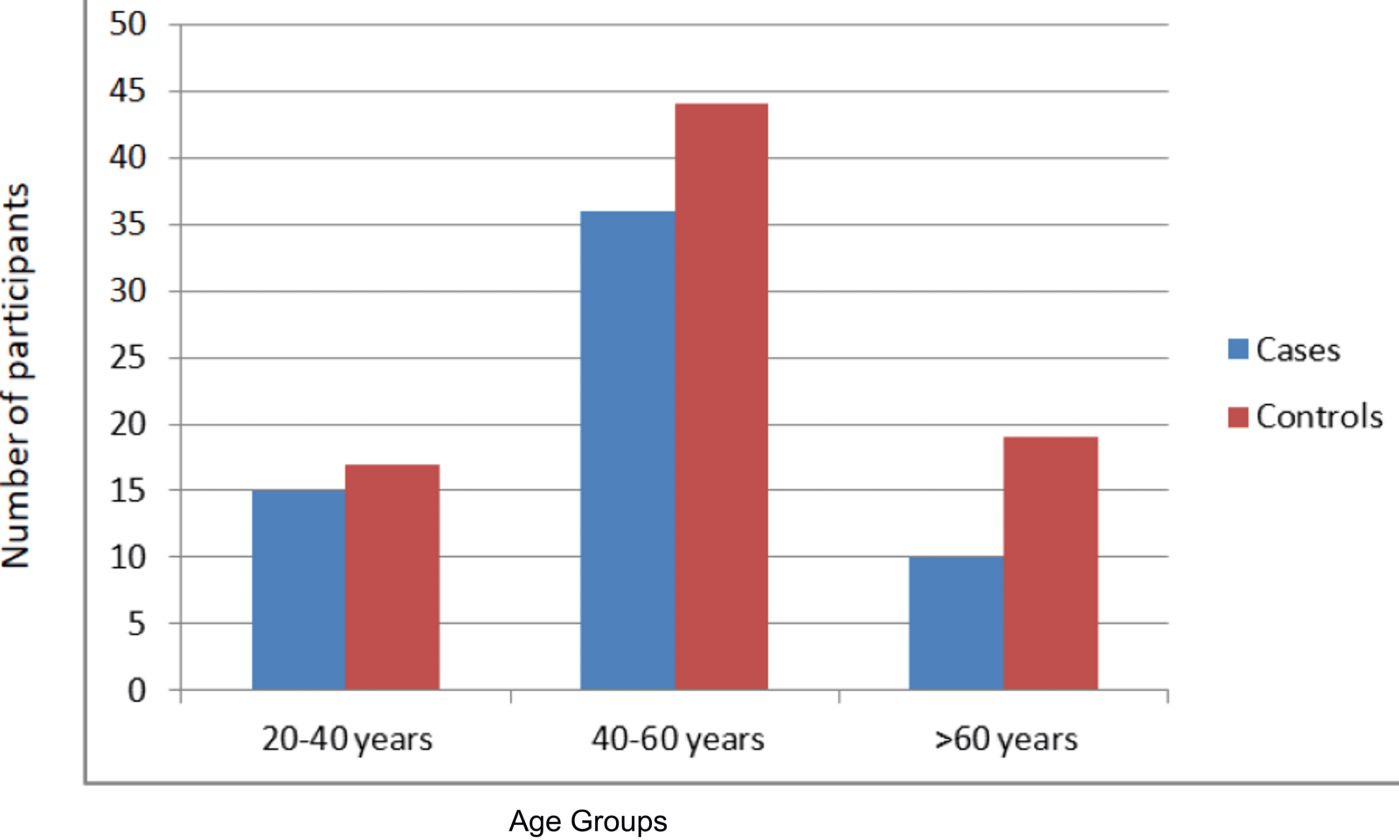
Age distribution of cases and controls.

The study included 120 participants divided into cases and controls according to fasting lipid profiles. Patients with deranged lipid parameters, such as elevated total cholesterol, low-density lipoprotein (LDL), or triglycerides, were categorised as cases (n = 60). Participants with normal lipid parameters served as controls (n = 60). Among the study population, 55 were female and 65 were male. All were above 20 years of age, with most between 40 and 60 years (Figure 2).

Primary analysis

**Figure 3:**
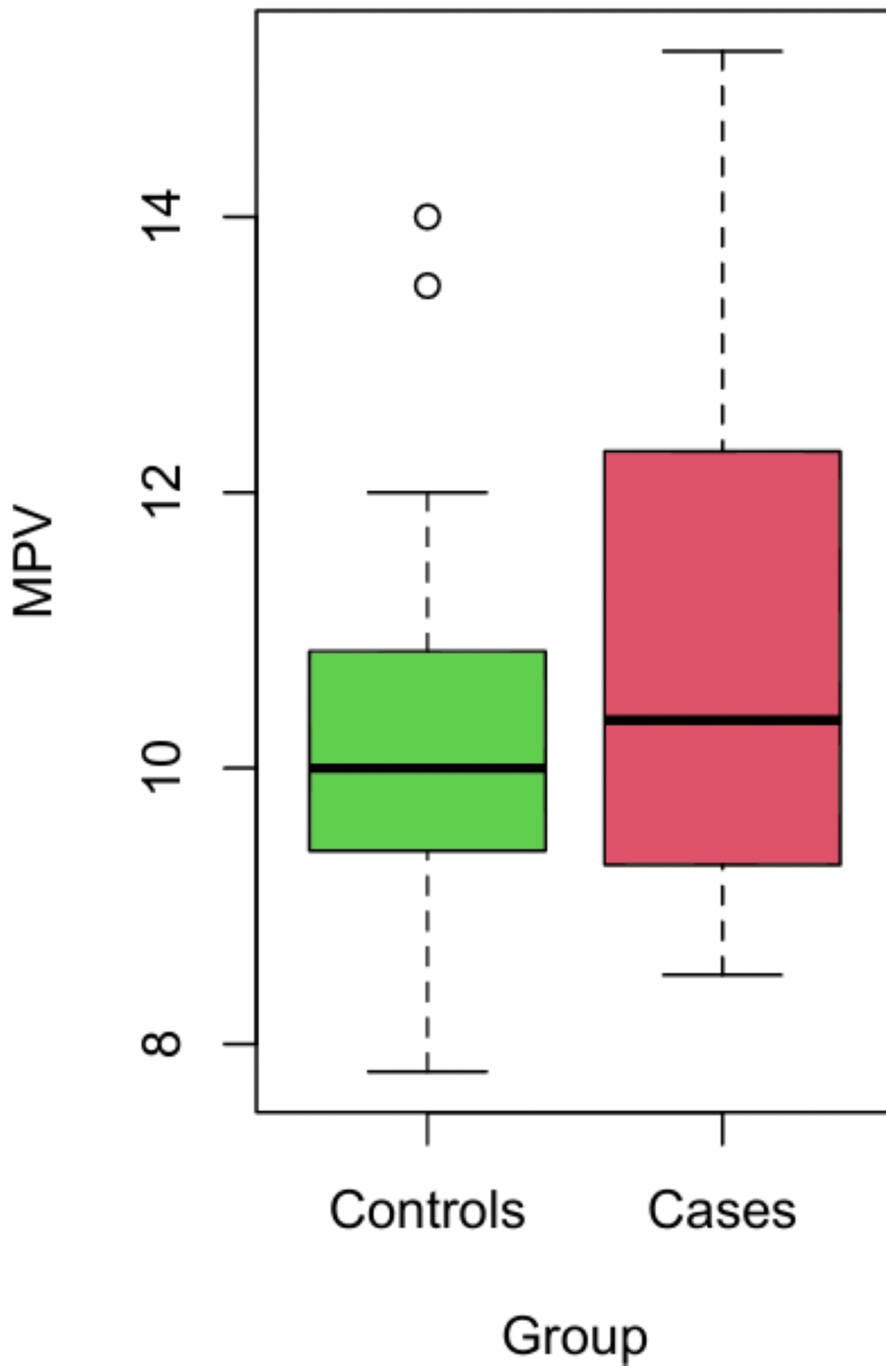
R-plot distribution of MPV MPV: mean platelet volume

**Figure 4:**
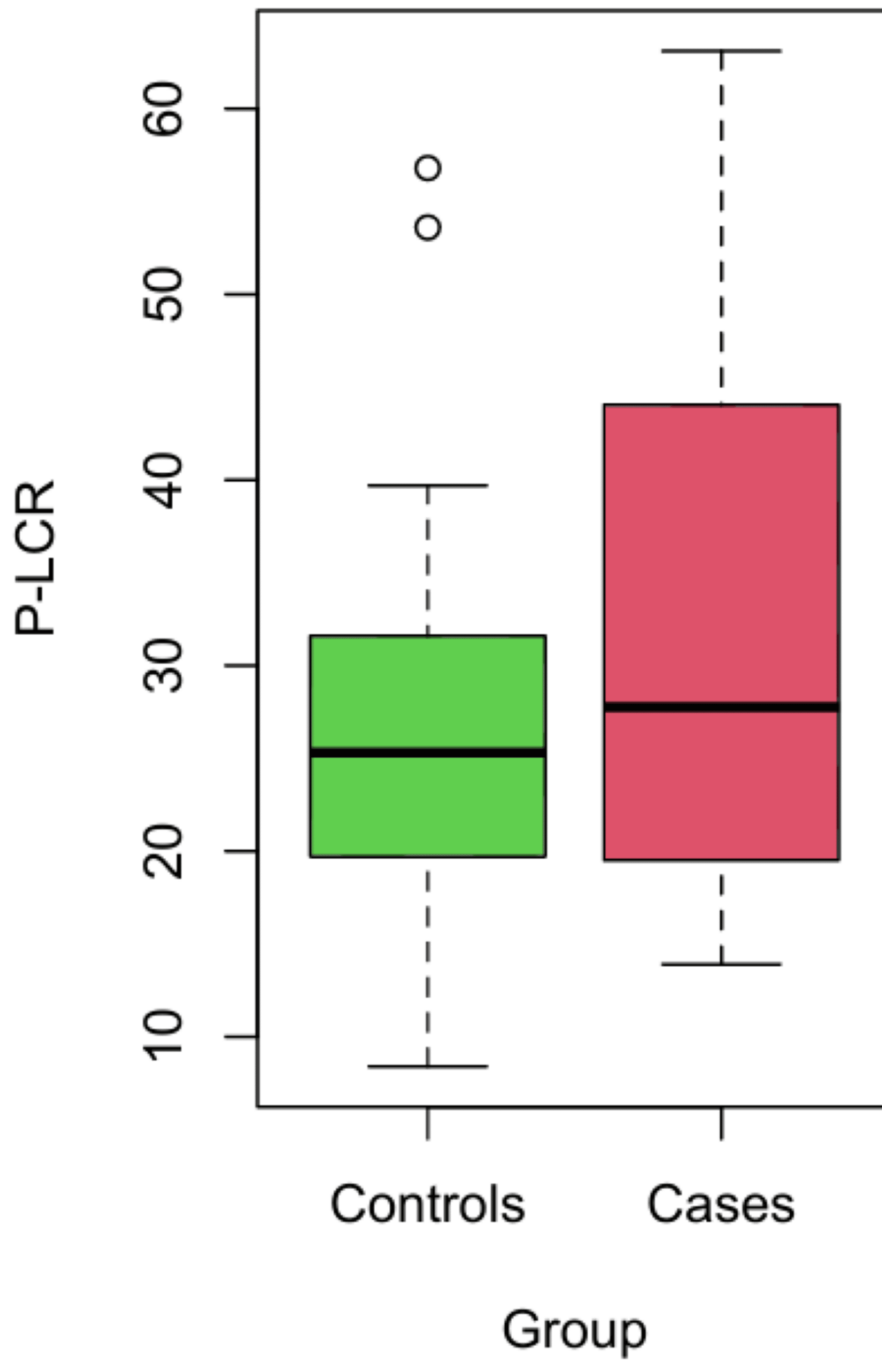
R-plot distribution of P-LCR P-LCR: platelet-large cell ratio

MPV, PDW, P-LCR, and Pct were compared between the case and control groups. As MPV was normally distributed, the mean value of cases and controls was compared using the independent t-test. The mean MPV was higher for cases than for controls, and the difference was statistically significant. As the variable P-LCR was not normally distributed, the median was compared using the Mann-Whitney U test. The median P-LCR value was higher in cases compared to controls, and the difference was statistically significant (p = 0.003 and p = 0.04, respectively) (Table 1, Figures 3, 4).

**Table 1:** . Statistical results for MPV and P-LCR *statistically significant MPV: mean platelet volume; P-LCR: platelet-large cell ratio

Parameter	Group	Mean ± SD	Median (IQR)	P-Value
MPV (fL)	Cases	10.97 ± 1.78	10.35 (3.00)	0.003*
	Controls	10.16 ± 1.13	10.00 (1.45)	
P-LCR (%)	Cases	32.69 ±14.11	27.75 (24.23)	0.04*
	Controls	26.07 ± 9.03	25.30 (11.90)	

After seeing that, in general, platelet parameters were increased among dyslipidaemic individuals than the control group, each platelet parameter was correlated with the lipid profile.

Correlation analysis

Triglyceride Levels

**Figure 5:**
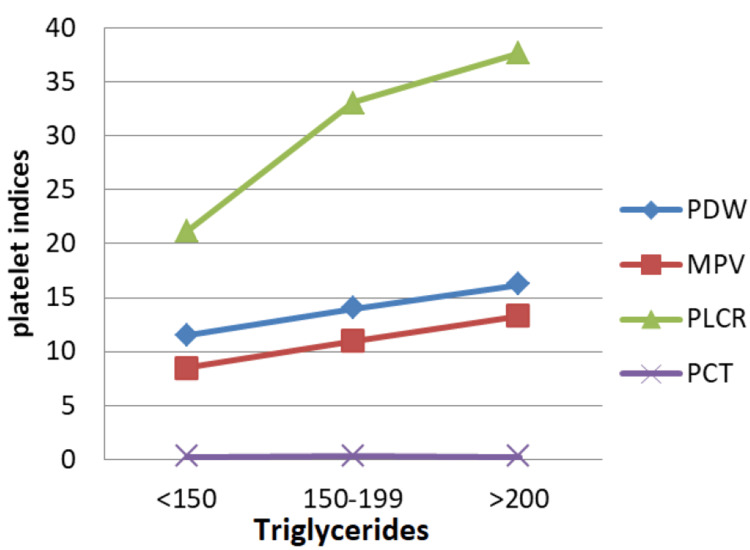
Correlation between PDW, MPV, P-LCR, and Pct and fasting triglyceride levels. Units: Triglycerides: mg/dL, PDW: fL, MPV: fL, P-LCR: %, and Pct: % PDW: platelet distribution width; MPV: mean platelet volume; P-LCR: platelet-large cell ratio; Pct: plateletcrit

According to the NCEP Adult Treatment Panel (ATP) III guidelines [13], fasting triglyceride levels are classified as normal (< 150 mg/dL), borderline high (150-499 mg/dL), high (200-499 mg/dL), and very high (> 500 mg/dL). Pct showed no marked change across these categories. However, P-LCR, MPV, and PDW exhibited upward trends, with P-LCR demonstrating a sharp rise when triglycerides exceeded 150 mg/dL (Figure 5).

**Figure 6:**
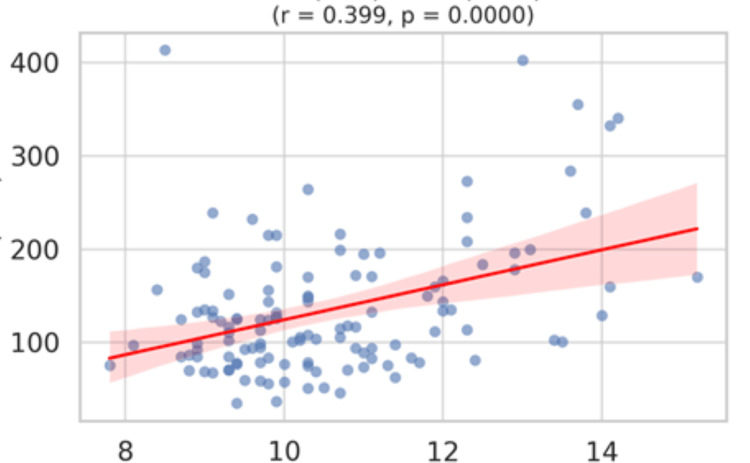
A scatterplot matrix (or pairwise correlation chart) for triglycerides vs MPV highlighting the significant correlation. Y axis: Triglycerides in mg/dL; X axis: MPV (mean platelet volume) in fL

By Pearson's correlation test, triglycerides showed a statistically significant positive correlation with MPV (r = 0.399, p < 0.001), P-LCR (r = 0.413, p < 0.001), and PDW (r = 0.468, p < 0.001). This implies that higher triglyceride levels are associated with larger and more heterogeneous platelets. A moderate positive correlation (r = 0.399, p < 0.001) shows that as triglyceride levels increase, mean platelet volume also tends to rise, indicating possible activation and enlargement of circulating platelets (Figure 6).

**Figure 7:**
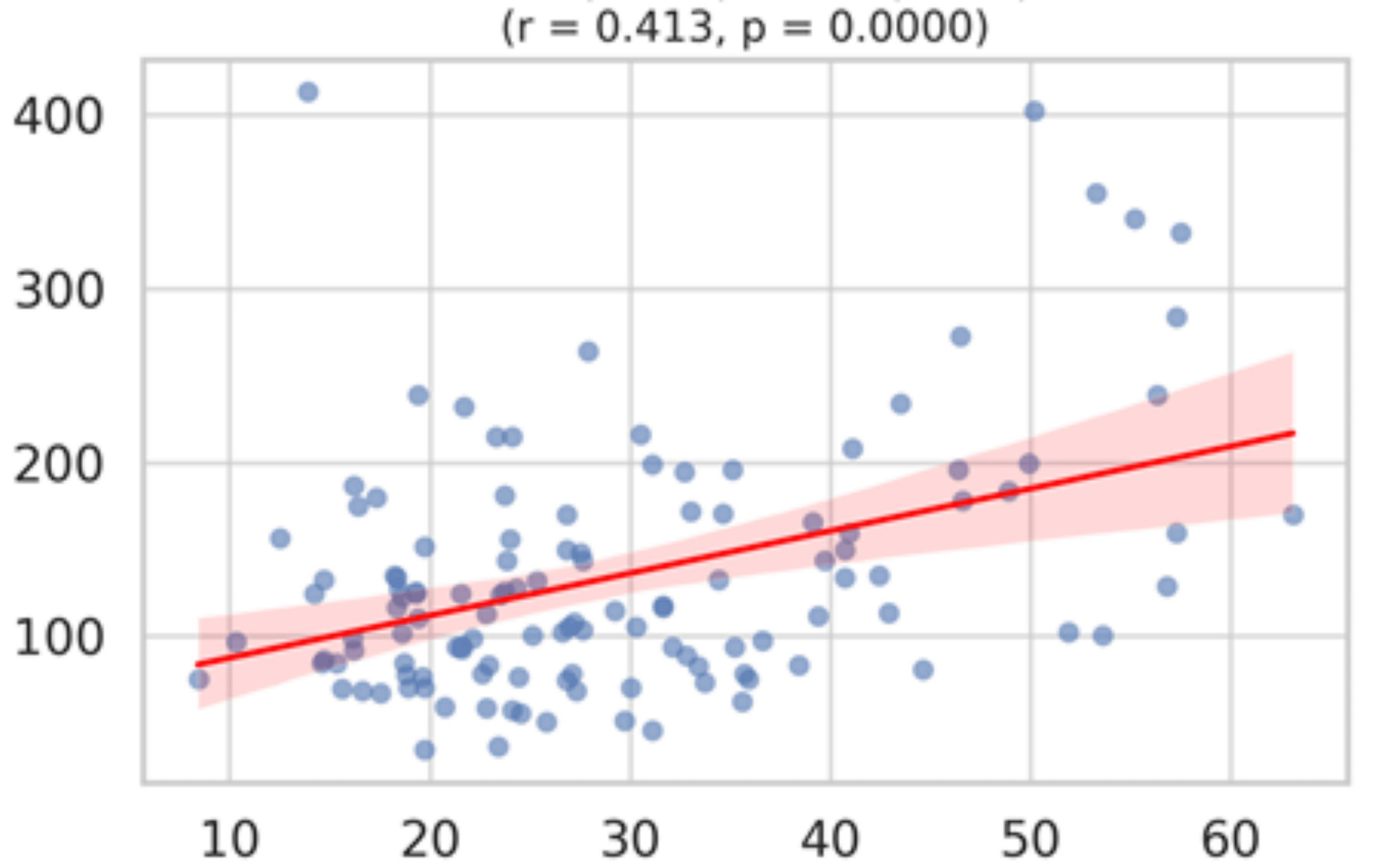
A scatterplot matrix (or pairwise correlation chart) for triglycerides vs P-LCR X axis: Platelet-large cell ratio (P-LCR) in %; Y axis: Triglycerides in mg/dL

Similarly, P-LCR demonstrates a significant positive association (r = 0.413, p < 0.001), confirming a higher proportion of large platelets with elevated triglycerides. (Figure 7).

**Figure 8:**
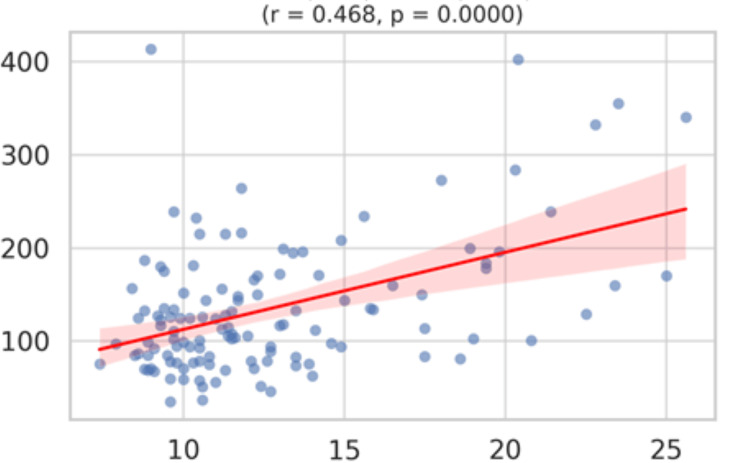
A scatterplot matrix (or pairwise correlation chart) for triglycerides vs PDW X axis: Platelet distribution width (PDW) in %; Y axis: Triglycerides in mg/dL

PDW exhibits the strongest relationship (r = 0.468, p < 0.001) among platelet indices, reflecting increased platelet heterogeneity with hypertriglyceridemia (Figure 8).

LDL Levels

**Figure 9:**
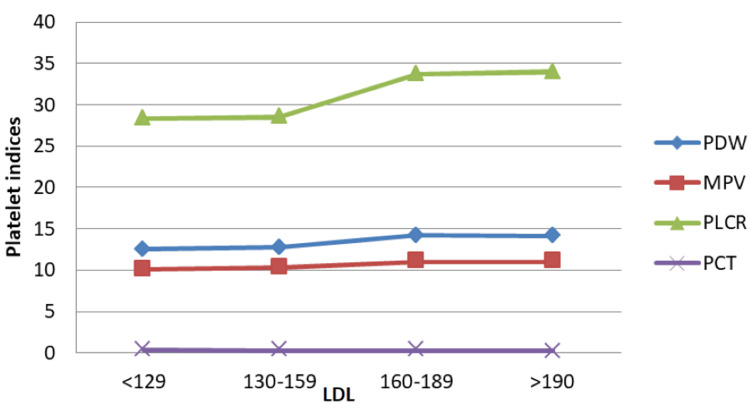
Correlation between PDW, MPV, P-LCR, and Pct and LDL levels. Units: LDL: mg/dL, PDW: fL, MPV: fL, P-LCR: %, Pct: % PDW: platelet distribution width; MPV: mean platelet volume; P-LCR: platelet-large cell ratio; Pct: plateletcrit; LDL: low-density lipoprotein

LDL was categorised as above-optimal (100-129 mg/dL), borderline-high (130-159 mg/dL), high (160-189 mg/dL), and very high (> 190 mg/dL), according to NCEP-ATP III criteria. Pct showed a mild downward trend with increasing LDL, while PDW and MPV fluctuated slightly. P-LCR increased progressively and displayed a marked rise at borderline-high LDL levels (Figure 9).

By Pearson's correlation test, LDL did not show any significant correlation with platelet indices (all p values > 0.05), suggesting limited direct association between cholesterol fractions and platelet morphology.

Total Cholesterol Levels

**Figure 10:**
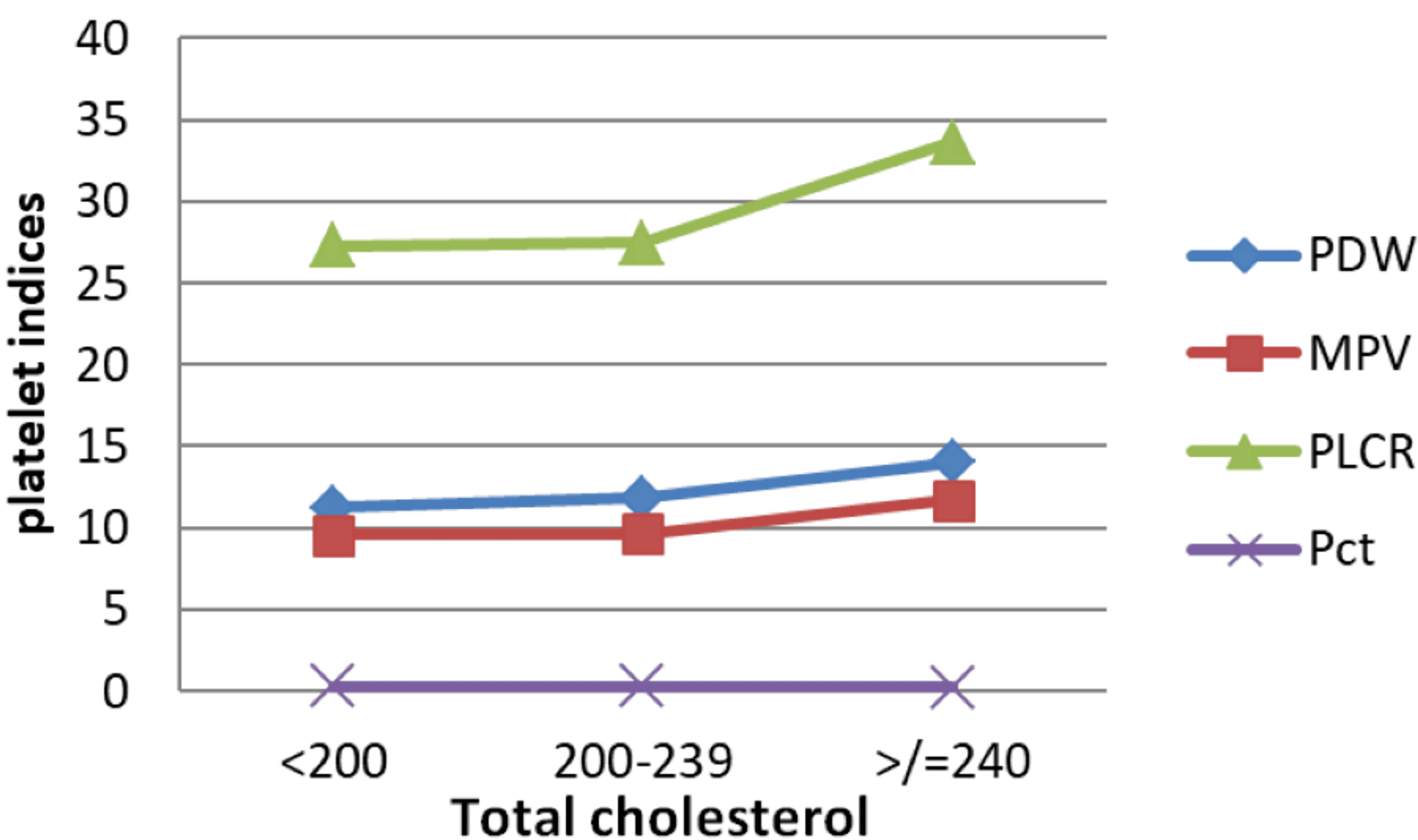
Correlation between PDW, MPV, P-LCR, and Pct and total cholesterol levels. Units: Total Cholesterol: mg/dL, PDW: fL, MPV: fL, P-LCR: %; Pct: % PDW: platelet distribution width; MPV: mean platelet volume; P-LCR: platelet-large cell ratio; Pct: plateletcrit

In line with NCEP guidelines [13], total cholesterol < 200 mg/dL is considered desirable, 200-239 mg/dL borderline-high, and ≥ 240 mg/dL high. Pct demonstrated a decreasing trend, whereas MPV, PDW, and P-LCR rose sharply in individuals with high cholesterol (Figure 10).

By Pearson's correlation test, total cholesterol did not show any significant correlation with platelet indices (all p values > 0.05), suggesting a limited direct association between cholesterol fractions and platelet morphology. On the whole, Pct remained independent of all lipid parameters, with near-zero correlations in Pearson's correlation test, indicating it behaves as a distinct platelet mass measure.

## Discussion

In this study, platelet indices were calculated and compared with lipid parameters, including total cholesterol, LDL, and triglycerides. The lipid parameters were classified according to the NCEP ATP III criteria [13]. The NCEP is a United States National Institutes of Health initiative aimed at reducing cardiovascular disease associated with hypercholesterolemia. The mean values were plotted as linear graphs, which demonstrated a marked increase in platelet indices corresponding to higher lipid levels. Platelet parameters (MPV, P-LCR, and PDW) were significantly higher in a proportion of cases compared with controls [6-12], whereas Pct showed no significant changes with a change in lipid levels. Sharma et al. compared PDW, P-LCR, and MPV and platelet function between patients with normal and abnormal lipid profiles, showing a statistically significant increase in all three indices among cases [14]. Our findings are consistent with those results, demonstrating similar elevations in these parameters. With respect to the association between lipid parameters and platelet indices, increases in MPV and P-LCR were proportionate to the levels of triglycerides and total cholesterol [15]. Several recent studies suggest that dyslipidemia promotes platelet activation and alters platelet indices through mechanisms such as oxidative stress and PI3K signalling. Gautam et al. also reported significant correlations between PDW and both LDL and total cholesterol in their study comparing platelet parameters and lipid profiles [8].

Several studies have reported that PDW and MPV are elevated in patients with valvular diseases and in those with abnormal lipid profiles [5,9]. A clinical study in the general population found that elevated MPV is associated with a higher risk of myocardial infarction, independent of established cardiovascular risk factors [10]. Similarly, Igor Spasic et al. [11] demonstrated that LDL levels are significantly associated with an increased risk of deep vein thrombosis. Statins remain the most commonly used hypolipidaemic agents. Various mechanisms have been proposed through which these drugs exert antiplatelet effects, and they may also act synergistically with other antiplatelet drugs such as clopidogrel [12].

When categorised according to the NCEP ATP III protocol [13], P-LCR exhibited a distinct plateau at borderline-high LDL and very high cholesterol levels. With respect to triglycerides, even minimal increases above the desirable range were associated with an increase in P-LCR, which continued to rise with higher triglyceride levels. Among the lipid parameters, P-LCR showed the most sensitive response to variations in fasting triglyceride concentration.

Limitations and future scope

In this study, most of the confounding factors, including acute inflammation, diabetes, smoking, use of hypolipidemic drugs, and antiplatelet drugs, are eliminated; thus, a real-time comparison can be done in the future with these individual confounding factors. Moreover, the relatively small sample size of our cohort is a critical limitation, restricting the generalizability of our findings to the broader population. Therefore, a larger community-based study is warranted for validation.

## Conclusions

The NCEP guidelines emphasise that cardiovascular morbidity risk is proportional to the severity of lipid elevation and recommend monitoring lipid levels to assess cardiovascular risk and to guide treatment initiation and dosage adjustment. Although fasting lipid profile tests are commonly used, they are relatively costly and require a fasting sample. In contrast, platelet parameters, which are inexpensive and readily available as part of a routine complete blood count, appear to be a promising, convenient, and cost-effective tool for monitoring patients with lipid disorders, as they do not require a fasting sample. However, given the preliminary nature of these findings, their definitive role in clinical practice requires validation in larger, population-based studies before they can be adopted as a standard monitoring tool.
